# Differentiation of primary lung cancer from solitary lung metastasis in patients with colorectal cancer: a retrospective cohort study

**DOI:** 10.1186/s12957-021-02131-7

**Published:** 2021-01-24

**Authors:** Jong Eun Lee, Won Gi Jeong, Yun-Hyeon Kim

**Affiliations:** 1 Department of Radiology, Chonnam National University Hospital, Chonnam National University Medical School, 42 Jebong-ro, Dong-gu, Gwangju, 61469 Republic of Korea; 2 Department of Radiology, Chonnam National University Hwasun Hospital, Hwasun-gun, Jeollanam-do Republic of Korea

**Keywords:** Solitary pulmonary nodule, Primary lung cancer, Solitary metastasis, Colorectal cancer, Computed tomography

## Abstract

**Background:**

This study aimed to evaluate the computed tomography (CT) features of solitary pulmonary nodule (SPN), which can be a non-invasive diagnostic tool to differentiate between primary lung cancer (LC) and solitary lung metastasis (LM) in patients with colorectal cancer (CRC).

**Methods:**

This retrospective study included SPNs resected in CRC patients between January 2011 and December 2019. The diagnosis of primary LC or solitary LM was based on histopathologic report by thoracoscopic wedge resection. Chest CT images were assessed by two thoracic radiologists, and CT features were identified by consensus. Predictive parameters for the discrimination of primary LC from solitary LM were evaluated using multivariate logistic regression analysis.

**Results:**

We analyzed CT data of 199 patients (mean age, 65.95 years; 131 men and 68 women). The clinical characteristic of SPNs suggestive of primary LC rather than solitary LM was clinical stages I–II CRC (*P* < 0.001, odds ratio [OR] 21.70). The CT features of SPNs indicative of primary LC rather than solitary LM were spiculated margin (quantitative) (*P* = 0.020, OR 8.34), sub-solid density (quantitative) (*P* < 0.001, OR 115.56), and presence of an air bronchogram (quantitative) (*P* = 0.032, OR 5.32).

**Conclusions:**

Quantitative CT features and clinical characteristics of SPNs in patients with CRC could help differentiate between primary LC and solitary LM.

**Supplementary Information:**

The online version contains supplementary material available at 10.1186/s12957-021-02131-7.

## Introduction

When a solitary pulmonary nodule (SPN) is detected in patients with colorectal cancer (CRC), differentiation between primary lung cancer (LC) and solitary lung metastasis (LM) can be crucial for treatment planning and predicting prognosis in clinical practice [1]. Moreover, surgical strategies for treating primary LC and solitary LM are quite different. In general, the treatment of choice for LM is minimally invasive surgical resection in order to preserve as much healthy lung parenchyma as possible in case repeat operation is needed. In contrast, complete surgical resection with lobectomy and mediastinal lymph node dissection is the gold standard for LC [2]. However, solitary LMs are more frequently reported in patients with CRC than in those with other extra-thoracic malignancies [3, 4], and primary LCs are occasionally reported to mimic solitary LMs [5, 6]. Therefore, it is sometimes difficult to determine whether a SPN is a primary LC or a solitary LM.

Image-guided needle biopsies may be useful for distinguishing between primary LC and solitary LM before surgical planning. However, it is difficult and risky to perform needle biopsies in some cases, especially for those with small lesions. Additionally, a small volume of biopsy specimen can impede histological differentiation between primary LC and solitary LM.

Imaging characteristics of SPN can be used as non-invasive alternatives to determine whether it is a primary LC or a solitary LM. However, compared to the generally accepted imaging findings of metastatic nodules including multiple peripherally located round variable-sized nodules [4], the comparison of imaging findings between primary LC and solitary LM is not well established. Therefore, the aim of this study was to determine the clinical characteristics and CT features that could be used to differentiate between primary LC and solitary LM in patients with CRC.

## Methods

### Patients

We retrospectively reviewed CRC patients by searching electronic medical records from January 2011 to December 2019 at a single tertiary referral center. Patients with the following criteria were included: presence of a SPN (defined as a round opacity in the lung, either well or poorly defined, measuring less than 30 mm [7]) on pre-diagnostic chest CT images, evidence of malignant potential such as size growth of a SPN that has increased in diameter of at least 2 mm, and availability of histopathologic report by thoracoscopic wedge resection. To this initial inclusion of 224 patients, we applied the exclusion criteria of patients whose SPN was not diagnosed as either primary LC or solitary LM (*n* = 13) and patients whose SPN deemed too small to characterize at pre-diagnostic chest CT image (less than 8 mm) (*n* = 12). Finally, 199 CRC patients were enrolled in this study (Table 1). Follow-up chest CT scans were obtained at 3, 6, 9, 12, 18, 24, 36, 48, and 60 months. Synchronous SPNs were defined as those occurring within 6 months of the diagnosis of CRC, while metachronous SPNs were defined as those occurring more than 6 months later [8]. After completing this 5-year follow-up program, follow-up chest CT scans were obtained every 2 years. The mean follow-up period and mean number of chest CT scans are summarized in Supplementary Table S1.

**Table 1:** Clinical characteristics of patients and SPNs

	LC (*n* = 70)	LM (*n* = 129)	*P* value
Age (years)	68.5 ± 8.15	64.6 ± 10.7	**0.004**
Male (years)	69.8 ± 6.56	65.3 ± 9.9	
Female (years)	66.3 ± 10.1	63.1 ± 12.2	
Sex (male/female)	44/26	87/42	0.515
History of smoking	37 (52.9)	49 (38)	**0.043**
Index tumor location			**0.003**
Colon	41 (58.6)	47 (36.4)	
Rectum	29 (41.4)	82 (63.6)	
Index tumor stage			**< 0.001**
Stages I–II	53 (75.7)	29 (22.5)	
Stages III–IV	17 (24.3)	100 (77.5)	
Chronicity of SPNs			**0.004**
Synchronous	18 (25.7)	13 (10.1)	
Metachronous	52 (74.3)	116 (89.9)	
Histopathology of SPNs		129 (100)	^++^N/A
Metastatic			
Adenocarcinoma	55 (78.6)		
Squamous cell carcinoma	14 (20)		
Small cell carcinoma	1 (1.4)		

Values in parentheses are percentages. Values are presented as mean ± standard deviation where applicable. Note: significant *P* values are shown in bold

*LC* lung cancer, *LM* lung metastases, *SPN* solitary pulmonary nodule, *CRC* colorectal cancer

^++^N/A, not applicable

### Histopathological diagnosis

Patients were divided into two groups based on histopathology: those with primary LC and those with solitary LM. Histopathological differentiation between primary LC and solitary LM was achieved by a board-certified thoracic pathologist with 15 years of experience. For histopathological differentiation, comprehensive histological assessment and immunohistochemistry staining including CK7, CK20, TTF-1, and CDX2 were performed. Nodules of different histological types including squamous cell carcinoma and small cell carcinoma were considered to be primary LC. Nodules with morphological features of pulmonary adenocarcinoma and positive staining for CK7 and TTF-1 were also considered to be primary LC. Nodules with morphological features of enteric adenocarcinoma and positive staining for CK20 and CDX2 were considered to be solitary LM [9, 10].

### Imaging protocols

Chest CT scans including high resolution CT images were obtained using the following multi-detector CT scanner: LightSpeed 16 (*n* = 87; GE Healthcare, Chicago, USA), LightSpeed VCT (*n* = 68; GE Healthcare, Chicago, USA), Somatom Definition Flash (*n* = 32; Siemens Healthineers, Erlangen, Germany), or Revolution (*n* = 11; GE Healthcare, Chicago, USA). For the LightSpeed VCT, LightSpeed 16, and Revolution, the following parameters were used: reconstruction thickness of the enhanced CT scan, 2.5 mm; rotation time, 0.5 to 0.8 s; peak kilovoltage, 120 kVp; and tube current, 60–220 mAs, with automatic exposure control. For the Somatom Definition Flash, the following parameters were used: reconstruction thickness, 2.5–3.0 mm; rotation time, 0.5 s; peak kilovoltage, 120 kVp; and tube current, 60–220 mAs, with automatic exposure control. Contrast-enhanced chest CT images were obtained after an intravenous injection of 120 to 130 mL nonionic contrast medium (either iohexol [Omnipaque®, Healthcare, Chicago, USA] or iopromide [Ultravist 300®, Bayer AG, Leverkusen, Germany]) at an average injection rate of 2 mL/s.

### Analysis of CT features

Chest CT images were interpreted independently by two thoracic radiologists with 20 and 8 years of experience, respectively. They were blinded to the clinical and histopathologic information of patients. If interpretations differed, a decision was made based on a consensus reading of two designated thoracic radiologists. If consensus was not achieved, the senior reader’s interpretation was accepted.

**Fig. 1:**
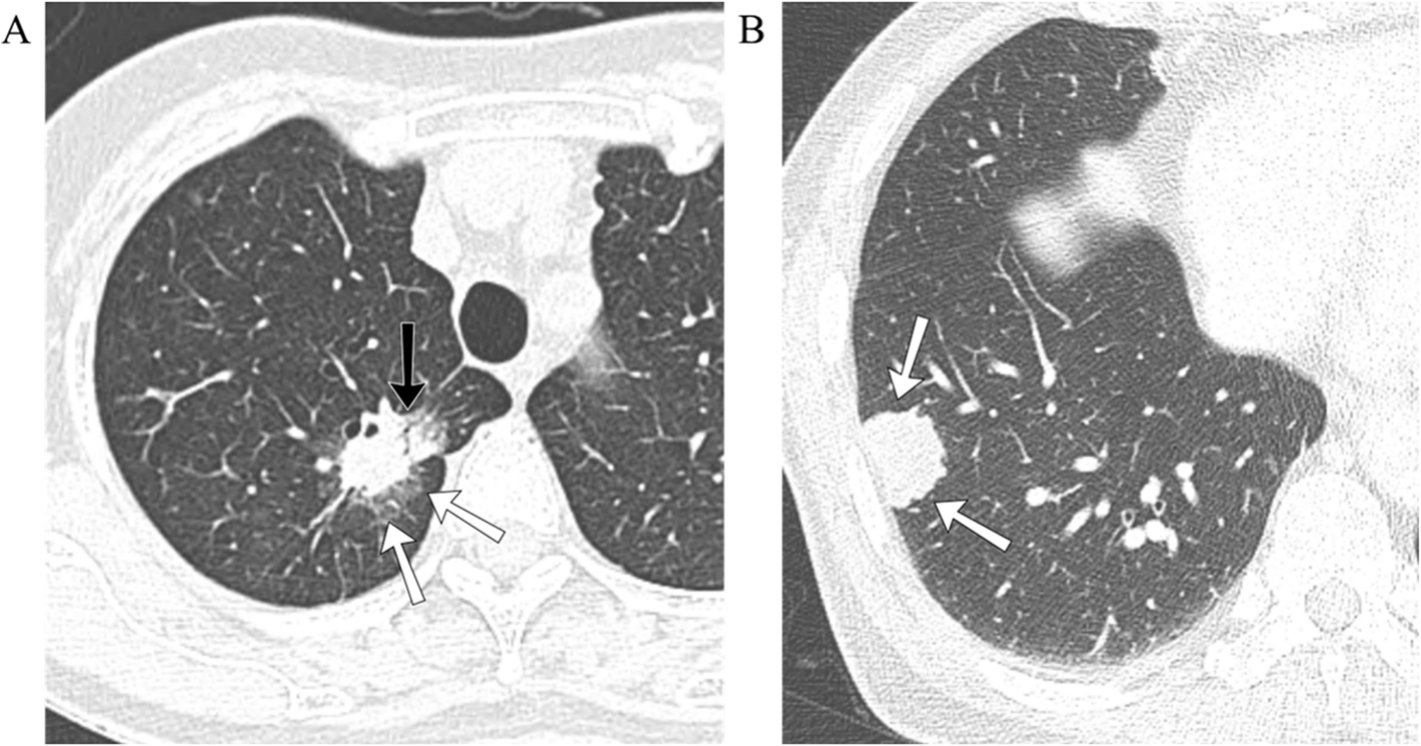
Computed tomography (CT) findings of primary lung cancer (LC) and solitary lung metastasis (LM). **a** Lung window image of contrast-enhanced chest CT scan showing a solitary nodule (white arrows) with sub-solid density, spiculated smooth margin, and presence of an air bronchogram (black arrow) in the right upper lobe. The nodule was histopathologically confirmed to be LC. **b** Lung window image of contrast-enhanced chest CT scan showing a solitary nodule (white arrows) with solid density and lobulated margin in the right lower lobe. The nodule was histopathologically confirmed to be LM

Qualitative CT features such as location (upper or non-upper, central or peripheral), margin (smooth, lobulated, or spiculated), and density (solid or sub-solid) of pulmonary nodules and presence of an air bronchogram, cavitation, pleural tags, pleural abutment, or background emphysema were assessed using chest CT images obtained with lung window settings (window width, 1500 HU; level, − 700 HU). A central location was defined as the area within 2 cm of the pulmonary hilum [11]. Nodules were classified as smooth, lobulated, or spiculated based on margin characteristics (Fig. 1a and b). Nodules were classified as having a sub-solid density if they contained a portion of ground-glass opacity (GGO) without completely obscuring bronchial or vascular margins of the lung parenchyma (Fig. 1a) [12]. An air bronchogram was defined as a gas-filled bronchus surrounded by abnormal lung parenchyma (Fig. 1a) [12]. Pleural tags were defined as linear strands that extended between nodule surface and adjacent pleural surface [12].

Quantitative CT features such as sizes of lung nodules were also assessed. The size of a nodule was measured using the longest diameter, including any portion of GGO seen on multiplanar reconstructed CT images (axial, coronal, and sagittal planes) obtained with lung window settings (window width, 1500 HU; level, − 700 HU) [13].

### Statistical analysis

All statistical analyses were performed using the SPSS software, version 25.0 (IBM, Armonk, USA). CT features of primary LC and solitary LM were compared using Pearson Chi-square test for categorical variables and independent *t* test for continuous variables. Post hoc analysis with Bonferroni’s correction was performed for multiple comparisons.

Inter-reader agreement for CT features was assessed by percent of concordant cases and kappa of agreement with 95% confidence intervals. A value of kappa lower than 0.20 was interpreted as poor agreement, 0.41–0.60 as moderate, 0.61–0.80 as substantial, and 0.81–1 as almost perfect agreement according to Cohen’s kappa coefficient [14]. Univariate and multivariate logistic regression analyses were used to evaluate the parameters predicting differentiation between the two groups. In initial univariate analysis, a *P* value of < 0.25 was used as the threshold for retaining factors in multivariate analysis [15]. Receiver operating characteristic (ROC) analysis was performed to evaluate the diagnostic ability to discriminate LC from LM according to each significant clinical characteristic and CT feature. Combined ROC curves were made using the predicted probability of significant independent factors. Corresponding area under the curve (AUC) was calculated, and comparisons between the AUCs were performed by the non-parametric approach of DeLong et al. [16]. Statistical significance was considered when *P* value was less than 0.05.

## Results

Clinical characteristics of patients enrolled in this study are summarized in Table 1. The mean age of patients was 65.9 ± 10 years. There were 131 men (mean age 66.8 ± 9.15 years) and 68 women (mean age 64.3 ± 11.5 years). In CRC patients, preoperative and surveillance chest CTs revealed 78 and 121 SPNs, respectively. The proportion of patients in which the index tumor was located in the rectum was significantly higher in the solitary LM group than that in the primary LC group (63.6% vs. 41.4%, *P* = 0.003). According to the American Joint Committee on Cancer tumor-node-metastasis staging system [17], the clinical stage of CRC patients were classified as I, II, III, and IV. The proportion of patients with clinical stages I–II index tumor was significantly higher in the primary LC group than that in the solitary LM group (77.5% vs. 24.3%, *P* < 0.001). The proportion of synchronous SPNs was significantly higher in the primary LC group than in the solitary LM group (25.7% vs. 10.1%, *P* = 0.004). CT features of SPNs were compared between primary LC and solitary LM groups (Table 2 and Supplementary Table S2). The mean size of nodules was significantly greater in the primary LC group (19.1 mm; IQR 15–22.5 mm) than in the solitary LM group (14.9 mm; IQR 10.0–17 mm) (*P* < 0.001).

**Table 2:** Comparison of CT features of SPNs

	LC (*n* = 70)	LM (*n* = 129)	*P* value
Size (mm)	19.1 ± 5.5	14.9 ± 6.2	**< 0.001**
Cranio-caudal location			0.188
Upper	35 (50.0)	52 (40.3)	
Non-upper	35 (50.0)	77 (59.7)	
Axial location			0.105
Central	12 (17.1)	12 (9.3)	
Peripheral	58 (82.9)	117 (90.7)	
Margin^a^			**< 0.001**
Smooth	7 (10)	54 (41.9)	
Lobulated	30 (42.9)	68 (52.7)	
Spiculated	33 (47.1)	7 (5.4)	
Density			**< 0.001**
Solid	47 (67.1)	128 (99.2)	
Sub-solid	23 (32.9)	1 (0.8)	
Air bronchogram	30 (42.9)	7 (5.4)	**< 0.001**
Cavitation	13 (18.6)	19 (14.7)	0.296
Pleural tags	41 (58.6)	25 (19.4)	**< 0.001**
Pleural abutment	32 (45.7)	53 (41.1)	0.528
Background emphysema	18 (25.7)	13 (10.2)	**0.004**

Values in parentheses are percentages. Values are presented as mean ± standard deviation where applicable. Size is a quantitative feature. Cranio-caudal location, axial location, margin, density, air bronchogram, cavitation, pleural tags, pleural abutment, and background emphysema are qualitative features

^a^Post hoc analysis was performed to compare the proportion of margin of SPNs between the two groups, smooth vs. lobulated, *P* = 0.005; smooth vs. spiculated, *P* < 0.001; lobulated vs. spiculated, *P* < 0.001. Significance level of 0.0167 takes into account the Bonferroni’s correction for post hoc analysis (0.05/3). Note: significant *P* values are shown in bold

*CT* computed tomography, *LC* lung cancer, *LM* lung metastases, *SPNs* solitary pulmonary nodules

The proportion of nodules with spiculated margins was significantly higher in the primary LC group than in the solitary LM group (47.1% vs. 5.4%, *P* < 0.001). The proportion of nodules with sub-solid density was significantly higher in the primary LC group than in the solitary LM group (32.9% vs. 0.8%, *P* < 0.001). Air bronchograms were significantly more frequent in the primary LC group than in the solitary LM group (42.9% vs. 5.4%, *P* < 0.001). Pleural tags were significantly more frequent in the primary LC group than in the solitary LM group (58.6% vs. 19.4%, *P* < 0.001). There were no statistically significant differences in the location of nodules or the presence of cavitation between the two groups (Table 2).

Inter-observer agreement for studied CT features was substantial (kappa 0.61–0.8) for central-peripheral location (kappa = 0.66), margin (kappa = 0.80), air bronchogram (kappa = 0.71), cavitation (kappa = 0.80), pleural tags (kappa = 0.80), and pleural abutment (kappa = 0.66). It was almost perfect (kappa 0.81–1) for all remaining CT features (Table 3).

**Table 3:** Analysis of inter-reader agreement showing the percent of concordance and kappa of agreement

CT features	Number (% of concordance)^a^	kappa (95% CIs)^b^
Cranial-caudal location	199/199 (100)	1 (1, 1)
Central-peripheral location	136/199 (68.3)	0.66 (0.50, 0.80)
Margin	174/199 (87.4)	0.80 (0.72, 0.87)
Density	192/199 (96.5)	0.83 (0.72, 0.95)
Air bronchogram	182/199 (91.5)	0.71 (0.58, 0.84)
Cavitation	188/199 (94.5)	0.80 (0.69, 0.91)
Pleural tags	180/199 (90.5)	0.80 (0.71, 0.88)
Pleural abutment	166/199 (83.4)	0.66 (0.55, 0.77)
Background emphysema	198/199 (99.5)	0.98 (0.94, 1.00)

*CI* confidence interval

^a^Values in parentheses are percentages

^b^Values in parentheses are 95% CIs

Predictive parameters for differentiation between primary LC and solitary LM were analyzed using univariate and multivariate logistic regression models (Table 4). Age (*P* = 0.009), history of smoking (*P* = 0.044), colon location of the index tumor (*P* = 0.009), clinical stages I–II CRC (*P* < 0.001), size of SPN (*P* < 0.001), spiculated margin (*P* < 0.001), lobulated margin (*P* = 0.007), sub-solid density (*P* ≤ 0.001), presence of an air bronchogram (*P* < 0.001), presence of pleural tags (*P* < 0.001), and background emphysema (*P* = 0.005) were identified as significant factors on univariate analysis. On multivariate analysis including these 13 factors as variables of interest, clinical stages I–II CRC (*P* < 0.001, odds ratio (OR) 21.70), spiculated margin (*P* = 0.020, OR 8.34), sub-solid density (*P* < 0.001, OR 115.56), and presence of an air bronchogram (*P* = 0.032, OR 5.32) were identified as significant independent factors for discriminating primary LC from LM.

**Table 4:** Multivariate analysis of clinical characteristics and CT features for discriminating LC from LM

	Univariate	*P* value	Multivariate	*P* value*
	OR		OR*	
Age	1.04 (1.01–1.08)	**0.009**	1.05 (0.99–1.11)	0.102
Smoking	1.83 (1.02–3.30)	**0.044**	2.81 (0.91–8.64)	0.072
Index tumor location				
Colon cancer	2.47 (1.36–4.48)	**0.009**	1.41 (0.52–3.85)	0.503
Rectal cancer	Reference		Reference	
Index tumor stage				
Stages I–II	10.75 (5.42–21.33)	**< 0.001**	21.70 (6.56–71.73)	**< 0.001**
Stages III–IV	Reference		Reference	
Size of SPN	3.34 (1.92–5.83)	**< 0.001**	2.01 (0.70–4.80)	0.197
Cranio-caudal location				
Upper	1.48 (0.82–2.66)	0.189	1.33 (0.46–3.79)	0.600
Non-upper	Reference		Reference	
Central location				
Central	0.50 (0.21–1.17)	0.110	2.11 (0.55–8.14)	0.280
Peripheral	Reference		Reference	
Margin				
Spiculated margin	36.37 (11.71–112.99)	**< 0.001**	8.34 (1.39–50.08)	**0.020**
Lobulated margin	3.40 (1.39–8.35)	**0.007**	2.41 (0.66–8.89)	0.186
Smooth margin	Reference		Reference	
Density				
Sub-solid density	62.64 (8.23–476.85)	**< 0.001**	115.56 (9.96–1341.06)	**< 0.001**
Solid density	Reference		Reference	
Air bronchogram				
Yes	13.07 (5.33–32.05)	**< 0.001**	5.32 (1.15–24.51)	**0.032**
No	Reference		Reference	
Cavitation				
Yes	1.32 (0.61–2.87)	0.482		
No	Reference			
Pleural tags				
Yes	5.88 (3.08–11.22)	**< 0.001**	2.41 (0.77–7.53)	0.131
No	Reference		Reference	
Pleural abutment				
Yes	1.21 (0.67–2.17)	0.529		
No	Reference			
Background emphysema				
Yes	3.06 (1.40–6.71)	**0.005**	1.83 (0.55–6.06)	0.322
No	Reference		Reference	

Data in parentheses are 95% confidence intervals. Each variable with a *P* value ≤ 0.25 in univariate analysis was analyzed in the multivariate model. All statistical analyses were performed using the logistic regression model. Note: significant ORs and *P* values are shown in bold

*CT* computed tomography, *LC* lung cancer, *LM* lung metastases, *OR* odds ratio

*Obtained by logistic regression model using all variables with a *P* value ≤ 0.25 in univariate analysis

**Fig. 2:**
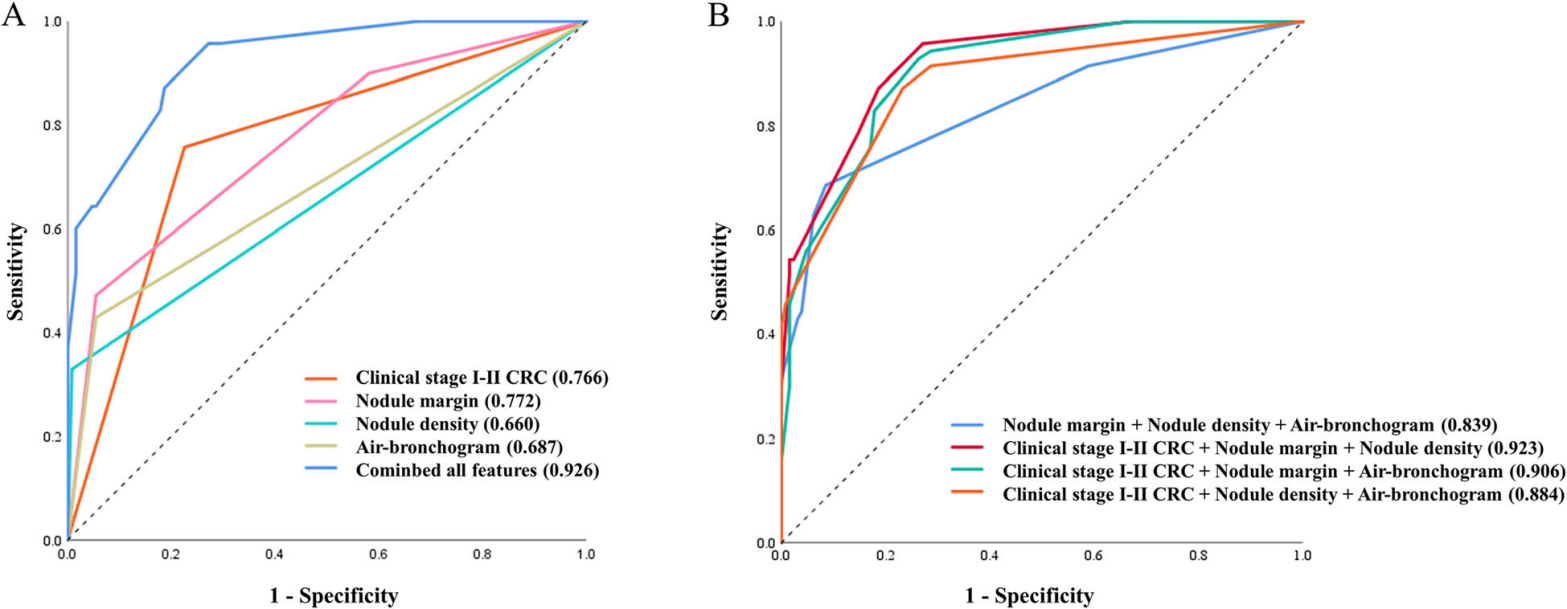
Receiver operating characteristic (ROC) curves for assessing the diagnostic ability of features to discriminate primary LC from solitary LM. **a** ROC curves for assessing the ability of features, both alone and in combination with all features, to discriminate primary LC from solitary LM. **b** ROC curves for assessing the ability of the combinations using 3 of all features to discriminate primary LC from solitary LM

ROC curves were used to assess the discrimination of primary LC from solitary LM using the 4 significant independent factors identified in multivariable logistic analysis. The AUCs of clinical stages I–II CRC, nodule margin, nodule density, and air bronchogram were 0.766, 0.772, 0.660, and 0.687, respectively (Fig. 2a). The AUC was 0.926 when all features were combined (Fig. 2a). Among all potential combinations using 3 of all features, the AUC significantly increased from 0.839 to 0.926 when clinical feature was added (*P* < 0.001) (Fig. 2b).

## Discussion

Marginal characteristics of nodules can be used to determine whether these nodules are primary or metastatic and whether they are benign or malignant [12, 18]. Previous studies have reported that a smooth or well-defined margin is more common in metastatic nodules than an irregular margin [4, 19]. In contrast, up to 80% of primary LC can present with a non-smooth margin, especially a spiculated margin which is already well-known to be associated with primary LC [12, 20, 21]. The proportion of nodules with spiculated margins was significantly higher in patients with primary LC than in patients with solitary LM in both univariate and multivariate analyses of our study. The margin of a nodule appeared more irregular even in solitary LM as the size increased [22]. However, solitary LM tended to show lobulated margin rather than spiculated margin in our study (Supplementary Table S2).

Nodules with a sub-solid density contain a GGO component commonly seen in lepidic growth of primary lung adenocarcinomas [23, 24]. Lepidic growth is defined as tumor progression along the alveolar wall. It is typically observed in primary lung adenocarcinomas. Only a few reports have described cases of lepidic growth of pulmonary metastases [25, 26]. Typically, pulmonary metastases present as solid, round nodules that are peripherally located [4]. In our study, sub-solid density of nodules was mostly observed in primary LC. It was rarely observed in solitary LM. Thus, sub-solid density of SPNs can be used to support the diagnosis of primary LC rather than that of solitary LM.

An air bronchogram is defined as an air-containing bronchus or bronchioles within an area of opacification of the surrounding alveoli. The presence of an air bronchogram within a nodule raises a high suspicion of a primary lung malignancy [12]. Air bronchograms have been reported to occur in primary LC of all histological types [27]. Only a few reports have described cases of pulmonary metastases showing air bronchograms [25]. The rate of air bronchograms within nodules was significantly higher in primary LC than in solitary LM in both univariate and multivariate analysis of our study.

Pleural tags are known as interlobular septal thickening of the lung between the nodule and visceral pleura. They may result from localized edema, tumor extension within or outside lymphatic vessels, inflammatory cells, or fibrosis [12]. A previous study has reported that pleural tags are commonly seen in primary LC and in up to 80% of surgically resected primary LC without abutting the pleura [28]. In the present study, pleural tags were found in 56.4% of primary LC. They were also significantly more frequent in primary LC than in solitary LM in univariate analysis of our study.

In addition to CT features, clinical characteristics can also aid the differentiation between primary LC and solitary LM. Several studies have previously characterized indeterminate pulmonary nodules in patients with CRC [29–32]. Among the factors predicting pulmonary metastasis, presence of lymph node metastasis in patients with CRC has been identified as a significant risk factor [29–32]. Kim et al. [33] have reported that the probability of pulmonary metastasis is low in patients with CRC without hepatic or lymph node metastasis, that is, in clinical stages I–II CRC patients. Similarly, the present study showed that solitary LM was associated with higher clinical stage (III–IV) CRC patients than lower clinical stage CRC patients (I–II) in both univariate and multivariate analyses.

Previous studies have reported that the location of the index tumor in the rectum rather than the colon is a risk factor of pulmonary metastasis in patients with CRC [29, 31]. The venous bloodstream of the rectum bypasses the liver, meaning that the first organ encountered is the lung [34]. Similarly, the proportion of index tumors located in the rectum was significantly higher in the solitary LM group than in the primary LC group in univariate analysis of the present study.

This study has several limitations. First, only nodules confirmed as either primary LC or solitary LM on histopathological analysis after surgical resection were included. There was an inherent selection bias towards patients who underwent surgery. Prospective studies (particularly randomized, controlled trials) are needed to confirm our results. Second, as this was a single-center and retrospective study, the sample size was relatively small. A study with a larger sample size is needed to validate our results. Third, visual analysis of CT features raises the possibility of inter-observer and intra-observer variability regarding categorization despite the use of consensus reading. For a more accurate interpretation, more quantitative analysis tool such as radiomics would be more helpful. Fourth, we did not consider other important information such as tumor metabolism or molecular information because it was not available in a substantial portion of our cases. Further researches would be needed in the future.

## Conclusion

CT features can be used to differentiate between primary LC and solitary LM. In our multivariate analysis, three CT features of nodules were found to be useful for differentiating primary LC and solitary LM. These were nodules with spiculated margin, sub-solid density, and presence of an air bronchogram. Understanding of the CT features of primary LC versus solitary LM allows better discrimination of SPNs in patient with CRC. Furthermore, both CT features of SPNs and clinical characteristics are needed to aid the differentiation between primary LC and solitary LM in CRC patients.

## Supplementary Information

**Additional file 1: Supplementary Table S1.** Characteristics of SPNs. **Supplementary Table S2.** Sub-group comparison of CT features of SPNs (≥20 mm)

## Data Availability

The study data is not available.

## Abbreviations

SPN: Solitary pulmonary nodule
CT: Computed tomography
CRC: Colorectal cancer
LC: Lung cancer
LM: Lung metastasis
OR: Odds ratio
GGO: Ground-glass opacity
ROC: Receiver operating characteristic
AUC: Area under the curve
