# Supplementary material for: Differentiation of primary lung cancer from solitary lung metastasis in patients with colorectal cancer: a retrospective cohort study
Source: World J Surg Oncol. 2021 Jan 24;19:28. doi: 10.1186/s12957-021-02131-7 (PMC7831192; doi:10.1186/s12957-021-02131-7)
Supplement: Supplementary file 1 — Additional file 1: Supplementary Table S1. Characteristics of SPNs. Supplementary Table S2. Sub-group comparison of CT features of SPNs (≥20 mm) [file 12957_2021_2131_MOESM1_ESM.docx]

| Supplementary Table S1. Characteristics of SPNs | | | |
| --- | --- | --- | --- |
|  | LC  (n=70) | LM  (n=129) | *P* value |
| Right versus left  Right upper lobe  Right middle lobe  Right lower lobe  Left upper lobe  Left lower lobe | 43/27 (61.4/38.6)  24 (34.6)  4 (5.7)  15 (21.4)  12 (17.1)  15 (21.4) | 66/63 (51.2/48.8)  15 (11.6)  11 (8.5)  40 (31.0)  35 (27.1)  28 (21.7) | 0.165 |
| Mean follow up (months) | 35.7 ± 37.3 | 29.8 ± 21.8 | 0.161 |
| Mean number of chest CT scans | 6.1 ± 3.7 | 6.3 ± 2.4 | 0.535 |
| Values in parentheses are percentages. Values are presented as mean ± standard deviation where applicable.  Note: significant *P* values are shown in bold.  CT, computed tomography. | | | |

| Supplementary Table S2. Sub-group comparison of CT features of SPNs (≥20 mm) | | | |
| --- | --- | --- | --- |
|  | LC  (n=39) | LM  (n=28) | *P* value |
| Size (mm) | 26.9 ± 6.5 | 25.0 ± 3.9 | 0.168 |
| Cranio-caudal location |  |  | 0.952 |
| Upper | 17 (43.6) | 12 (42.9) |  |
| Non-upper | 22 (56.4) | 16 (57.1) |  |
| Axial location |  |  | 0.105 |
| Central | 9 (23.1) | 6 (21.4) |  |
| Peripheral | 30 (76.9) | 22 (78.6) |  |
| Margin^*^ |  |  | **0.001** |
| Smooth | 2 (5.1) | 4 (14.3) |  |
| Lobulated  Spiculated | 16 (41.0)  21 (53.8) | 21 (75.0)  3 (10.7) |  |
| Density |  |  | **0.005** |
| Solid | 27 (69.2) | 27 (96.4) |  |
| Sub-solid | 12 (30.8) | 1 (3.6) |  |
| Air-bronchogram | 18 (46.2) | 2 (7.1) | **0.001** |
| Cavitation | 6 (15.4) | 4 (14.3) | 0.296 |
| Pleural tags | 28 (7.18) | 10 (35.7) | **0.003** |
| Pleural abutment | 21 (53.8) | 16 (57.1) | 0.789 |
| Background emphysema | 10 (25.6) | 3 (10.7) | 0.128 |
| Values in parentheses are percentages. Values are presented as mean ± standard deviation where applicable. Size is a quantitative feature. Cranio-caudal location, axial location, margin,density, air-bronchogram, cavitation, pleural tags, pleural abutment, and background emphysema are qualitative features.  ^*^Post-hoc analysis was performed to compare the proportion of margin of SPNs between the two groups, smooth vs. lobulated, *P* = 0.648; smooth vs. spiculated, *P* = 0.005; lobulated vs. spiculated, P = 0.001. Significance level of 0.0167 takes into account the Bonferroni's correction for post-hoc analysis (0.05/3).  Note: significant *P* values are shown in bold.  CT, computed tomography; LC, lung cancer; LM, lung metastases; SPNs, solitary pulmonary nodules | | | |
